# The Expanding Role of the Urologist in Metastatic Prostate Cancer: From Biopsy to Surgical Interventions

**DOI:** 10.5041/RMMJ.10564

**Published:** 2026-01-28

**Authors:** Nicola Fazaa, Etan Eigner, Ameer Nsair, Melissa Atallah, Gilad Amiel, Azik Hoffman

**Affiliations:** The Department of Urology, Rambam Health Care Campus, Haifa, Israel

**Keywords:** Cytoreductive radical prostatectomy, metastatic prostate cancer, prostate biopsy

## Abstract

The evolving landscape of metastatic prostate cancer (mPCa) necessitates a redefinition of the urologist’s role, extending beyond diagnosis to active participation in therapeutic and surgical management. This review outlines evidence-based approaches to biopsy and surgical interventions across the disease spectrum. Prostate biopsy remains fundamental for diagnosis, treatment stratification, and molecular profiling, with targeted and metastatic lesion sampling improving precision oncology. For symptom relief, surgical management of bladder outlet obstruction through transurethral resection of the prostate and holmium laser enucleation of the prostate remains essential, with emerging data suggesting possible oncologic benefits when combined with systemic therapy. GreenLight photoselective vaporization may represent an alternative option, though evidence remains limited. Cytoreductive radical prostatectomy in carefully selected patients with metastatic hormone-sensitive disease may provide improved local control and delayed progression, supported by growing biological rationale but constrained by the retrospective nature of current evidence. Collectively, these findings underscore the expanding multidisciplinary role of the urologist in mPCa care, emphasizing the need for prospective studies to validate the integration of surgical approaches within systemic treatment frameworks.

## INTRODUCTION

Prostate cancer remains a global health challenge, with nearly 1.5 million new cases and approximately 400,000 deaths reported worldwide in 2022.^1^ Metastatic disease accounts for a significant proportion of this burden, with incidence rates projected to rise by 1.03% annually through 2025, driven in part by declining prostate-specific antigen (PSA) screening rates following revised guidelines.^2^,^3^ In the United States alone, more than 300,000 newly diagnosed patients and 35,000 deaths are anticipated in 2025, underscoring the urgency of optimizing management strategies.^2^

A shift in the epidemiology of metastatic prostate cancer (mPCa) characterized by younger age at diagnosis and persistent racial disparities demands a reevaluation of the traditional urological paradigms.^3^ Traditionally, systemic therapy has been the cornerstone of management for mPCa, with palliative interventions addressing symptoms such as bladder outlet obstruction (BOO). However, emerging evidence suggests that surgical interventions, including holmium laser enucleation of the prostate (HoLEP), transurethral resection of the prostate (TURP), and cytoreductive radical prostatectomy, may offer select patients improved quality of life and, in some cases, potential survival benefits. While approaches to mPCa differ internationally, particularly regarding the adoption of cytoreductive surgery and prostate-specific membrane antigen-guided biopsy, common trends emphasize multidisciplinary integration and patient selection. The urologist, once primarily involved in diagnosis and localized disease treatment, now plays a broader role in managing patients across the entire disease spectrum. This paper examines evidence-based approaches to diagnosis and surgical intervention in mPCa, emphasizing the evolving diagnostic and therapeutic responsibilities of the urologist.

## PROSTATE BIOPSY

Prostate biopsy plays a pivotal role in the management of mPCa, serving as a cornerstone for confirming the diagnosis, characterizing tumor biology, and guiding personalized therapeutic strategies. While biopsies of the primary tumor are a well-established standard in localized prostate cancer, their application in the metastatic setting is increasingly recognized, particularly in the context of advances in imaging technologies and molecular profiling. In many cases, biopsy remains essential when imaging findings are inconclusive or when histopathological confirmation is required to initiate systemic therapy.^4^ Additionally, tissue acquisition is often necessary to enable genomic analyses that inform precision medicine approaches.

In patients presenting with significantly elevated PSA levels, particularly those exceeding 75 ng/mL, a limited biopsy protocol may be sufficient. Evidence suggests that a two-core prostate biopsy can detect metastatic disease with diagnostic accuracy of up to 97.9% while reducing complications (*P*=0.003) and pain scores (*P*=0.03) compared to standard protocols, thereby reducing the risk of complications associated with traditional systematic 12-core sampling protocols.^4^

Moreover, biopsies of metastatic sites, such as osseous or soft-tissue lesions, are increasingly used not only for diagnostic confirmation but also to identify actionable genetic alterations and histologic variants. For instance, the detection of neuroendocrine differentiation may prompt a shift from androgen deprivation therapy to platinum-based chemotherapy, reflecting the aggressive biology of treatment-emergent neuroendocrine prostate cancer.^5^

While systematic biopsies, typically involving 12 to 14 cores from various regions of the prostate, remain a diagnostic mainstay, targeted biopsy approaches are gaining preference in the metastatic setting.^4^ These targeted biopsies utilize advanced imaging modalities such as multiparametric magnetic resonance imaging and prostate-specific membrane antigen positron emission tomography, which enhance lesion detection and facilitate more precise tissue sampling, thereby improving diagnostic yield and reducing unnecessary tissue acquisition.^4^

Biopsies of metastatic lesions also offer critical insights into tumor evolution and mechanisms of therapeutic resistance. Molecular profiling of metastatic tissue can reveal clinically relevant alterations such as *BRCA2* or *ATM* mutations, which predict sensitivity to poly(ADP-ribose) polymerase inhibitors, or androgen receptor gene amplifications associated with resistance to second-generation androgen-receptor-targeted therapies such as enzalutamide or abiraterone.^5^,^6^

Furthermore, the integration of emerging technologies, such as prostate-specific membrane antigen positron emission tomography, has significantly enhanced the accuracy of lesion localization for metastatic biopsy procedures. These advances improve diagnostic precision and minimize sampling error. Artificial intelligence-driven imaging platforms are also under investigation for their potential to predict biopsy success and optimize patient selection by analyzing lesion-specific radiomic features. In clinical practice, the choice between limited, targeted, or metastatic site biopsy should be guided by disease extent, PSA level, and the need for molecular characterization, using a patient-centered, resource-appropriate approach. In practice, a sequential approach can be applied: (1) confirm diagnosis through targeted or limited prostate biopsy depending on PSA and imaging findings; (2) assess symptom burden and relieve BOO as appropriate; and (3) evaluate eligibility for cytoreductive surgery in selected patients based on disease burden, performance status, and systemic therapy response.

## SURGICAL MANAGEMENT OF URINARY RETENTION

Urinary retention is a significant and distressing complication in patients with advanced or mPCa, affecting approximately 13% of cases and substantially impacting quality of life.^7^ It can arise due to tumor infiltration, direct compression of the urethra, or coexisting benign prostatic hyperplasia-related adenoma, leading to lower urinary tract symptoms, hydronephrosis, and even renal dysfunction if left untreated.^8^,^9^ To alleviate symptoms and improve urinary flow, surgical interventions are often required. Among the available procedures, TURP and HoLEP are commonly considered for relieving BOO.^7^,^8^,^10^

Palliative TURP, often referred to as “channel” or “tunnel TURP,” has been selectively utilized in patients with advanced prostate cancer to manage BOO-related symptoms. Although the current evidence is primarily derived from retrospective cohort studies, these reports consistently demonstrate that TURP can effectively relieve BOO and improve quality of life, with a relatively low risk of perioperative complications in appropriately selected patients.^11^–^13^

Importantly, some recent studies also suggest a potential oncological benefit. For instance, palliative TURP and photoselective vaporization of the prostate (PVP) have been associated with improved cancer-specific survival in men with mPCa when compared to androgen deprivation therapy alone, raising the possibility that cytoreductive local therapy may play a beneficial role in systemic disease management.^9^,^14^

Overall, palliative TURP remains a clinically relevant option in the multidisciplinary management of urinary retention in mPCa. With proper selection and integrated systemic therapy, TURP may provide not only effective palliation but also potential survival benefits in selected patients.

Holmium laser enucleation of the prostate is an emerging alternative to traditional TURP for patients with advanced prostate cancer who experience urinary retention or severe BOO. It is particularly indicated for patients with high prostate volume or those who still have significant symptoms following pharmacological treatment or androgen deprivation therapy.^10^,^15^ There are several potential advantages of HoLEP over conventional TURP, such as reduced bleeding, shorter catheterization time, and the ability to remove larger volumes of prostatic tissue in a single procedure.^10^

The minimally invasive nature of HoLEP makes it an appealing option for patients with advanced disease who may be less fit for larger surgical interventions due to limited physiological reserves. Studies evaluating HoLEP in patients with advanced prostate cancer have consistently shown significant improvements in urinary parameters.^15^ In a retrospective analysis involving 38 patients with advanced prostate cancer and urinary retention, patients treated with HoLEP in combination with complete androgen blockade demonstrated faster, more substantial, and lasting improvements in International Prostate Symptom Score, Quality of Life scores, maximal flow rate, and post-void residual volume compared to patients receiving complete androgen blockade alone. The benefits of HoLEP were sustained over an 18-month follow-up period, suggesting provision of durable symptomatic relief.^15^ Similarly, a study of 28 patients with advanced prostate cancer (≥cT3) reported significant improvements in mean total International Prostate Symptom Score, Quality of Life score, maximal flow rate, and post-void residual volume at 1 month post-HoLEP, with these benefits maintained through 12 months of follow-up.^10^

Although HoLEP has demonstrated considerable efficacy, it is associated with a higher risk of complications in patients with advanced prostate cancer. Transient urinary incontinence is a common postoperative concern, with reported incidence rates of up to 50% at 1 month and 21.43% at 3–12 months following surgery. Key factors associated with early transient urinary incontinence (at 1 month) include lower preoperative maximal flow rate, larger preoperative post-void residual volume, and bladder neck tumor invasion. Older age and longer enucleation time were identified as significant predictors of persistent transient urinary incontinence at 3–12 months.^10^,^15^ Multivariate analysis has shown that preoperative maximal flow rate and bladder invasion are independent predictors of postoperative incontinence. In addition, HoLEP may be associated with a higher risk of additional perioperative complications such as bleeding (though less frequent than in traditional TURP), urinary tract infections, and bladder neck contractures. Still, HoLEP generally demonstrates a favorable safety profile, with relatively rare serious perioperative or postoperative events reported in most studies.^10^,^15^

An alternative to conventional monopolar or bipolar TURP/HoLEP is GreenLight™ PVP (GreenLight™ Laser System; Boston Scientific, Marlborough, MA, USA). In a retrospective series of 39 advanced prostate cancer patients with acute urinary retention, GreenLight HPS 120-W PVP was found to be safe and provided functional improvements (in International Prostate Symptom Score, peak flow rate, postvoid residual) at 1, 3, 6, and 12 months postoperatively, with a mean PSA nadir of 0.33 ng/mL and a low rate of severe complications. The authors suggested that early palliative vaporization might provide some degree of tumor cytoreduction in addition to symptom relief.^14^ However, this is a small, uncontrolled study, and oncological outcomes are not robustly evaluated. Compared to TURP and HoLEP, evidence on GreenLight PVP in the context of mPCa is very limited. Other newer modalities, e.g. Aquablation (Aquablation® therapy; AquaBeam® Robotic System, PROCEPT BioRobotics, Redwood City, CA, USA) and Rezūm (Rezūm™ water vapor thermal therapy system; Boston Scientific, Marlborough, MA, USA), similarly lack prospective or long-term data in mPCa. Thus, while GreenLight may be an option in select patients, its role should currently be considered experimental and adjunctive until more rigorous data emerge.

Moreover, open simple prostatectomy, whether by suprapubic or retro-pubic approach, is less commonly reported in the context of mPCa due to its invasive nature and the availability of less invasive alternatives. The current literature lacks substantial evidence regarding the efficacy and safety of open prostatectomy specifically for urinary retention in mPCa patients. Therefore, its role remains limited and should be considered on a case-by-case basis, taking into account the patient’s overall health status and disease progression.

A comparative summary of the surgical approaches discussed is presented in Table 1.

**Table 1:** Summary of Surgical Options for Management of Bladder Outlet Obstruction in Metastatic Prostate Cancer.

Procedure	Indications	Advantages	Limitations/Considerations
TURP	Used for urinary retention relief or obstructive symptoms in locally advanced or metastatic disease	Well-established, widely available, and provides rapid symptom relief	May require re-intervention; limited evidence on oncologic impact in the metastatic setting
HoLEP	Applied for BOO or urinary retention, particularly in larger glands or in patients under systemic therapy	Associated with reduced bleeding risk and durable symptom control	Requires specific expertise; limited prospective data in metastatic prostate cancer
GreenLight PVP	Considered a minimally invasive option for patients unfit for TURP or HoLEP	Provides functional improvement with a favorable safety profile	Evidence remains limited; oncologic benefit not yet established

BOO, bladder outlet obstruction; HoLEP, holmium laser enucleation of the prostate; PVP, photoselective vaporization of the prostate; TURP, transurethral resection of the prostate.

## CYTOREDUCTIVE RADICAL PROSTATECTOMY

Cytoreductive radical prostatectomy (CRP) might demonstrate oncological benefits in metastatic hormone-sensitive prostate cancer (mHSPC) but is based on relatively small cohorts reported in prospective and retrospective trials. In the Local Treatment of Metastatic Prostate Cancer trial, CRP patients (*n*=17) achieved 100% 2-year overall and cancer-specific survival compared with 61% and 55% in patients treated with standard care (*n*=29), respectively.^16^ Larger analyses show CRP reduces cancer-specific mortality (HR 0.62, 95% confidence interval [CI] 0.43–0.89) and all-cause mortality (HR 0.58, 95% CI 0.36–0.93) compared to systemic therapy alone.^17^

The general concept of cytoreductive surgery is well established and has demonstrated oncologic benefit in several malignancies, including ovarian, gastrointestinal, and renal cell carcinomas, in which reduced primary tumor burden contributes to improved systemic disease control and survival.^18^–^21^ Translating this paradigm to mPCa, CRP aims to decrease overall tumor load, potentially alter tumor–host interactions, and modulate the immune microenvironment. Experimental and translational data suggest that removal of the primary tumor may reduce circulating tumor cell dissemination, improve response to systemic therapy, and delay the onset of castration resistance. Recent reports have also high-lighted possible immunologic effects of CRP, including modulation of systemic inflammatory markers and enhancement of systemic therapy efficacy.^22^

However, the increase in treatment burden and possible higher risk of permanent urinary incontinence should be discussed with the patient. Interestingly, the IP5-MATTER study quantifies patient preferences, revealing a willingness to accept 10% increased urinary incontinence risk for a 3.4-month survival gain.^23^ Still, functional outcomes remain acceptable, with 29.4% of CRP patients experiencing stress urinary incontinence versus 6.8% urge incontinence in non-surgical groups.^17^ Major complications (Clavien–Dindo ≥3) were reported to occur in 5% of cases, contrasting with 33% local progression rates in androgen deprivation therapy-only patients.^17^,^24^ While CRP delays castration resistance (40 versus 29 months in androgen deprivation therapy cohorts),^25^ these findings derive from non-randomized studies with selection bias toward younger patients and lower metastatic burden.^25^

Despite these encouraging results, randomized controlled trials are needed to confirm the survival benefits of CRP and to identify the optimal patient population for this approach. As new treatments for mPCa are introduced, extending the time to cancer-specific mortality in these patients, re-considering the option of surgical primary disease site intervention is warranted, as current evidence suggests that patients with low metastatic burden and good performance status may derive the greatest benefit from CRP. Ongoing studies will help clarify the role of CRP as part of a multimodal treatment strategy for mHSPC, potentially improving outcomes while maintaining acceptable quality of life.

However, while this biological rationale is compelling, the clinical evidence remains largely retrospective and heterogeneous. In contrast to other metastatic malignancies, randomized controlled data supporting CRP in mPCa are still lacking. Additionally, unlike metastatic renal cell carcinoma, where cytoreductive nephrectomy has established roles, the benefit of CRP is not yet definitively proven, and ongoing prospective trials are expected to clarify its utility. These limitations underscore the importance of careful patient selection and multidisciplinary evaluation when considering CRP in the metastatic setting.

This review is limited by the retrospective nature of most available data, selection bias in surgical cohorts, and the absence of large randomized controlled trials evaluating CRP or palliative surgical interventions. Further prospective studies are needed to validate these findings.

## CONCLUSION

As the epidemiology of mPCa continues to evolve, so too must the role of the urologist. This review highlights how urologists are increasingly engaged across the full continuum of care from diagnostic biopsy to symptom-directed surgery and, in selected cases, cytoreductive intervention.

Advanced biopsy strategies, including targeted and metastatic site sampling, have become essential not only for diagnosis but also for genomic profiling and treatment stratification. Surgical management of BOO through procedures such as TURP and HoLEP remains a cornerstone of palliative care, with emerging data suggesting potential oncologic benefit when combined with systemic therapy. Moreover, the concept of CRP reflects an expanding frontier in mPCa management, supported by biological rationale and early evidence but limited by retrospective data and selection bias.

Future directions should prioritize prospective randomized trials to better define patient selection criteria, clarify survival benefits, and integrate local surgical therapy within multimodal systemic strategies. Ultimately, the expanding scope of urological practice in mPCa underscores the need for multidisciplinary collaboration, individualized treatment planning, and continued research to refine the balance between oncologic control and quality of life.

## Abbreviations

BOO: bladder outlet obstruction
CRP: cytoreductive radical prostatectomy
HoLEP: holmium laser enucleation of the prostate
mPCa: metastatic prostate cancer
PSA: prostate-specific antigen
PVP: photoselective vaporization of the prostate
TURP: transurethral resection of the prostate.
